# The Endochitinase of *Clonostachysrosea* Expression in *Bacillus amyloliquefaciens* Enhances the *Botrytis cinerea* Resistance of Tomato

**DOI:** 10.3390/ijms19082221

**Published:** 2018-07-30

**Authors:** Yangyang Zheng, Xudong Wang, Siyuan Liu, Kewei Zhang, Zhibo Cai, Xiuling Chen, Yao Zhang, Jiayin Liu, Aoxue Wang

**Affiliations:** 1 College of Life Sciences, Northeast Agricultural University, Harbin 150030, China; zhengyangyang625@163.com (Y.Z.); 13552607994@163.com (X.W.); dabaozicaizhibo@163.com (Z.C.); zy13263696020@163.com (Y.Z.); 2 Key Laboratory of Biology and Genetic Improvement of Horticultural Crops (Northeast Region), Ministry of Agriculture/Northeast Agricultural University, Harbin 150030, China; liusiyuan6992@126.com (S.L.); zhangkewei225@163.com (K.Z.); xiuling.chen@ymail.com (X.C.); 3 College of Sciences, Northeast Agricultural University, Harbin 150030, China; 13040216@163.com

**Keywords:** *Bacillus amyloliquefaciens*, chitinase, defense enzymes, biological control

## Abstract

To investigate whether the *ech42* gene in *Clonostachysrosea* can improve the biocontrol efficacy of *Bacillus amyloliquefaciens* and its molecular mechanism. Compared to the wild type, the *B. amyloliquefaciens* transformed with the ech42 gene exhibited higher chitinase activity. The *B. amyloliquefaciens*-ech42 also showed significantly higher biocontrol efficiency compared to *Botrytiscinerea* when tomato plants were pre-treated with *B. amyloliquefaciens-*ech42. No significant difference in biocontrol efficiency was observed between the wild type and *B.amyloliquefaciens-*ech42 when tomato plants were first infected by *Botrytiscinerea*. In addition, the activity of the defense-related enzyme polyphenol oxidase, but not superoxide dismutase, was significantly higher in *B. amyloliquefaciens-*ech42 than in the wild type. The ech42 enhances the biocontrol efficiency of *B.amyloliquefaciens* by increasing the capacity of preventative/curative effects in plants, rather than by killing the pathogens.

## 1. Introduction

Biological control (biocontrol) of phytopathogens provides an attractive alternative means of managing plant diseases [1]. It is highly efficient and causes no harm to the environment or to human health. *Clonostachysrosea* is a fungus commonly found in the environment and non-pathogenic to plants. It is a plant probiotic, which belongs to the family Moniliaceae [2]. *C. rosea* is a common soil fungus, with a wide range of hosts [3], able to withstand adverse environments [4]. The fungus *C.rosea* has been successfully tested as a biocontrol species against many plant pathogenic fungi, and has shown great potential in controlling plant diseases and promoting crop growth [5,6,7,8,9]. *C. rosea* inhibits the growth of pathogenic fungi mainly by producing extracellular lytic enzymes, especially chitinase, which can degrade chitin, the main component of fungal cell walls [10,11]. Other studies have shown that chitinase mayenhance the inhibition of many plant pathogens [12,13,14,15].

The antifungal effect of chitinase was first reported by Horikoshi and Iida, who found that *Bacillus circulans* exerted lytic activity against *Aspergillusoryzae,* and that the addition of chitinase increased the lytic activity [16,17]. Over-expression of the chitinase gene (chit36 or chit42 of *Trichodermaharzianum* and cht42 of *T. virens*) enhanced the inhibition to *Botrytiscinerea* [12,13,14,15].

Another well-studied biocontrol agent is *Bacillus amyloliquefaciens*, which inhibits plant pathogens by producing low molecular weight antibiotics and other active substances such as *B.amyloliquefaciens* antibiotic production [18]. *B. amyloliquefaciens* can also promote plant growth [19]. Purification of chitinases from *B. amyloliquefaciens* has also been reported [14,20], and these chitinases displayed antifungal activities.

In this study, in an effort to further enhance the antifungal activity of *B. amyloliquefaciens*, the endochitinase gene of *C. rosea* (encoded by ech42), was transformed into *B. amyloliquefaciens*. The biocontrol effects of *B. amyloliquefaciens*-ech42 in vitro against *Botrytiscinerea* were investigated and our results provided evidence of substantially improved inhibitory effects on *Botrytiscinerea*. 

## 2. Results

### 2.1. Construction of pHT43-ech42

Digestion of the plasmid pHT43-ech42 by *Xba* I and *Sma* I showed that the pHT43 vector and the *ech42* gene were about 7000 and 1300 bp respectively, which matched the expected sizes. The results of single and double digestion showed that the recombination vectors of pTH43-ech42 were constructed successfully (Figure S1).

### 2.2. Transformation of ech42 Gene to B. amyloliquefaciens

For electroporation transformation, different voltages were used to test the transformation efficiency. The highest number of transformants was obtained at 15 kv/cm and no transformants were obtained at 20 kv/cm or 21 kv/cm, probably due to the increase in cell death.

### 2.3. Detection the Growth Curves of B. amyloliquefaciens and Recombinant B. amyloliquefaciens Strains

The growth rate of recombinant strain (*B. amyloliquefaciens*-ech42) was slower than the wild type (*B. amyloliquefaciens*). The difference in growth rates gradually increased after 14 h culture and reached a maximum after 24 h (Figure S2). 

### 2.4. Detection of Recombinant ech42 Protein by SDS-PAGE

**Figure 1:**
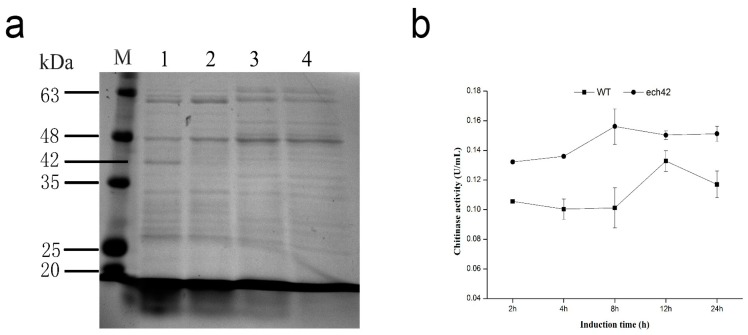
ech42 protein expression analysis of *B. amyloliquefaciens* and *B. amyloliquefaciens*-ech42. (**a**) SDS-PAGE showing the ech42 protein from *B. amyloliquefaciens*-ech42. M. Protein Marker; (1) *B. amyloliquefaciens-*ech42 induced by IPTG; (2) *B. amyloliquefaciens*-ech42 without IPTG induction; (3) *B. amyloliquefaciens* induced by IPTG; (4) *B. amyloliquefaciens* without IPTG induction; (**b**) Comparison of chitinase activities of *B. amyloliquefaciens* and *B. amyloliquefaciens*-ech42.

SDS-PAGE showed a 42 kDa protein band in *B. amyloliquefaciens*-ech42 in the presence of IPTG. However, no similar band was observed in *B. amyloliquefaciens*-ech42 in the absence of IPTG or in *B. amyloliquefaciens* induced with IPTG (Figure 1a). The 42 kDa band was similar to the molecular mass of ech42, indicating that the ech42 was successfully expressed in the *B. amyloliquefaciens*-ech42 strain. 

### 2.5. Chitinase Activity of B. amyloliquefaciens and B. amyloliquefaciens-ech42

A chitinase activity assay showed that the *B. amyloliquefaciens-ech42* displayed significantly higher chitinase activity than the wild type strain. The activity of *B. amyloliquefaciens*-ech42 was highest at the 8 h incubation (0.156 ± 0.012 U/mL), which was 1.54 times higher than that of the wild type strain (0.101 ± 0.014 U/mL) (Figure 1b). The activity of the wild type was highest at 12 h (0.133 ± 0.007 U/mL), 1.13 lower than the activity of *B. amyloliquefaciens-ech42* (0.15 ± 0.003 U/mL) at the same time point. 

### 2.6. Biocontrol Efficiency of B. amyloliquefaciens-ech42 vs. Botrytiscinerea in Greenhouse

**Figure 2:**
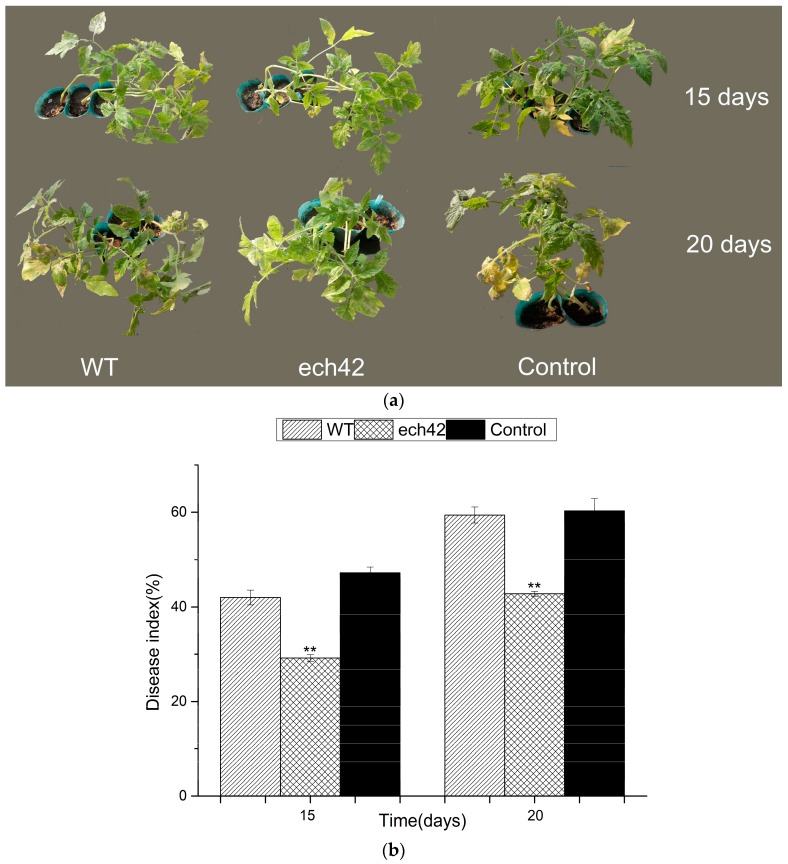

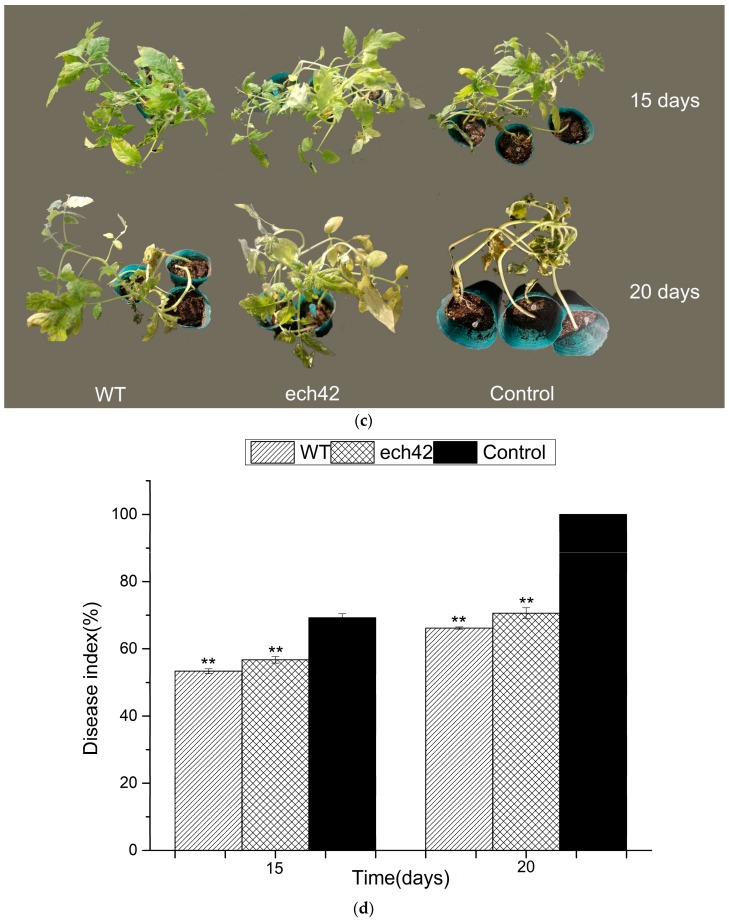
The biocontrol efficiency of *B. amyloliquefaciens*-ech42 against *Botrytiscinerea* on tomato plants. The prevention experiment (**a**,**b**): (**a**) Representative images of tomato plants treated with wild type *B. amyloliquefaciens* (WT), *B. amyloliquefaciens*-ech42 (ech42) or non-treated (control); (**b**) disease indexs in the prevention experiment at 15 and 20 days. The treatment experiment (**c**,**d**): (**c**) Representative images of tomato plants treated with wild type *B. amyloliquefaciens* (WT), *B. amyloliquefaciens*-ech42 (ech42) or non-treated (control); (**d**) disease index in the treatment experiment at 15 and 20 days. Differences between the treatment and control were analyzed using ANOVA and significant results are indicated by ** (*p* < 0.01).

The biocontrol efficiency of *B. amyloliquefaciens*-ech42 against *Botrytiscinerea* was further tested using tomato plants in a greenhouse setting. In the prevention experiment (Figure 2a,b), the *B. amyloliquefaciens*-ech42 exhibited significantly higher biocontrol efficiency, compared to the wild type *B. amyloliquefaciens,* at both day 15 and day 20. However, in the treatment experiment, no significant differences in biocontrol efficiency were observed between the wild type and *B. amyloliquefaciens*-ech42 (Figure 2c,d). These results suggest that the increased chitinase activity in *B. amyloliquefaciens* works more efficiently in plant pathogen prevention than in pathogen treatment. 

### 2.7. Changes in Defense-Related Enzyme Activity

**Figure 3:**
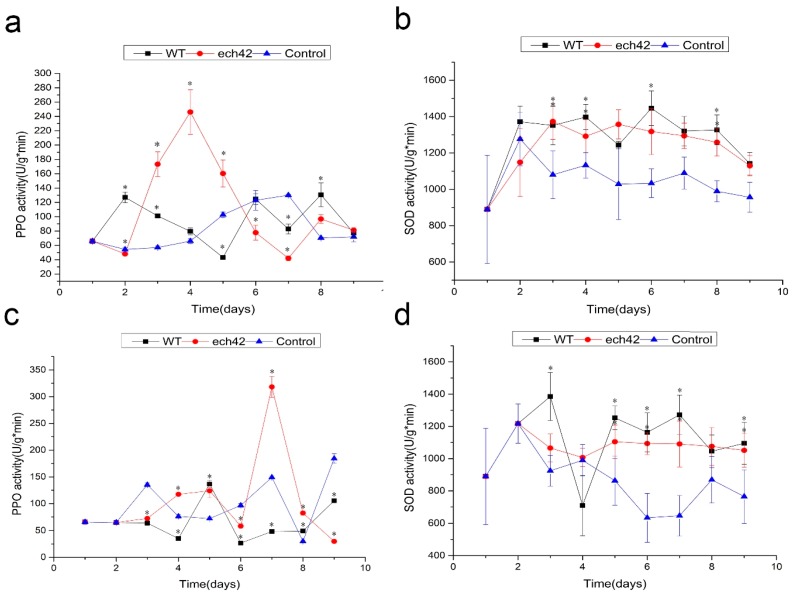
Changes of PPO (**a**,**c**) and SOD (**b**,**d**) activities during prevention (**a**,**b**) and treatment (**c**,**d**) experiments against *Botrytiscinerea* on tomato plants. WT: *B. amyloliquefaciens*; ech42: *B. amyloliquefaciens-*ech42; control: not treated with either WT or ech42. “*”: indicates statistically significant results.

During the prevention and treatment experiments, fresh leaves were collected from all treatment groups to test the enzyme activities of both PPO and SOD. In the prevention experiments, the activities of PPO with *B. amyloliquefaciens-*ech42 were significantly higher than those of *B. amyloliquefaciens* or the control (days 3–5). Compared to *B. amyloliquefaciens* or the control, *B. amyloliquefaciens-*ech42 displayed higher PPO activity in the treatment experiment at day 7 (Figure 3a,c). The SOD activities of both the wild type and *B. amyloliquefaciens-*ech42 were significantly higher than those of the control group, but no significant differences were observed between the wild type and *B. amyloliquefaciens*-ech42 (Figure 3b,d). 

## 3. Discussion

In this study, we transformed the endochitinase *ech42* gene isolated from *C. rosea* into the biocontrol bacterium *B. amyloliquefaciens*. We then compared the biocontrol efficiency between the wild type and the *B. amyloliquefaciens* over expressing the chitinase. Consistent with previous studies, we showed that the increased chitinase in *B. amyloliquefaciens*-ech42 was effective for inhibiting the growth of *Botrytiscinerea*. We also showed that *B. amyloliquefaciens*-ech42 was more efficient in plant pathogen prevention than in pathogen treatment.

The transformation efficiency of plasmid was affected by several factors, with the main factor being applied voltages. In this study, the highest number of pHT43-ech42 transformants were obtained with 15 kv/cm, and no transformants were obtained at 20 kv/cm or 21 kv/cm. This is consistent with a previous study, which showed the transformation frequency was 12 kV/cm, and that subsequently, transformation frequency reduced with increased voltage, probably due to the increase in cell death [21]. 

In this study, we found that the growth rate of *B. amyloliquefaciens*-ech42 was lower than that of the wild type. In contrast, a previous study showed that the growth rate of a recombinant strain was higher than that of a wild type of *Streptomyces* sp [17]. The authors speculated that over expression of chitinase could degrade chitin (the solocarbon source) into glucose, further supporting cell growth [15].

To further understand the molecular mechanism of the ech42 enhanced biocontrol effect, we investigated the activities of two defense-related enzymes, PPO and SOD. PPO is an oxidase and catalyzes the oxidation of monophenols and o-diphenols to o-diquinones, which can ultimately produce brown or red pigments that cause fruit browning. PPO also plays important roles in disease resistance. It has been shown that over expression of PPO increased resistance to the bacterial pathogen, *Pseudomonas syringae* [22]. SOD acts as an antioxidant that catalyzes the dismutation of reactive oxygen species, which cause oxidative stress and damage to cells [1]. In this study, we found that the PPO and SOD activities of *B. amyloliquefaciens*-ech42 were significantly higher than those of the wild type, when tomato plants were treated first with *B. amyloliquefaciens*-ech42 (prevention experiment). However, when the plants were treated first with the plant pathogen *Botrytiscinerea*, the PPO and SOD activities were not significantly higher. Our results strongly suggest that chitinase of *B. amyloliquefaciens-*ech42 enhances the biocontrol efficiency by increasing the prevention capacity of plants, rather than by killing the pathogens. As a result, a good strategy for effective biocontrol is to apply the biocontrol agents as early as possible to boost the “immune system” of plants.

One limitation of our study is that our results do not confirm whether the enhanced biocontrol efficiency of the recombinant *B. amyloliquefaciens* (*B. amyloliquefaciens*-ech42) is caused primarily by the increased level of chitnase. It is possible that the insertion of ech42 may cause other changes to *B. amyloliquefaciens*. Studies using purified chitinase are needed to further understand the biocontrol mechanism of *B. amyloliquefaciens.*

In conclusion, our study demonstrated that insertion of the *C. rosea* endochitinase gene ech42 into *B. amyloliquefaciens* significantly enhanced the chitinase activity and its biocontrol efficiency. Our results suggest that the increased biocontrol efficiency may be caused by the increased capacity of preventative/curative effects in plants, rather than by killing the pathogens.

## 4. Materials and Methods

### 4.1. Strains and Culture Conditions

Bacterial and fungal strains used in this study are listed in Table S2, and the bacterial strains *B. amyloliquefaciens* were isolated from the fruits of tomato plants. *B. amyloliquefaciens* and *C. rosea* were cultured in Luria broth (LB) medium (1% tryptone, 1% NaCl and 0.5% yeast extract) at 37 °C. Fungi were grown on a potato dextrose agar (PDA) plate (20% potatoes (sliced, washed, unpeeled), 2% dextrose and 2% agar).

### 4.2. Construction of ech42 Plasmid

*C. rosea* was cultured in a PDA plate. RNA was extracted and reverse transcribed into cDNA, which was used as the DNA template. The primers for the *C. rosea ech42* gene were designed based on the sequence from the GenBank: DQ523687 (Table S1). The *ech42* gene was amplified by a polymerase chain reaction (PCR). The PCR mixture included: 1 µL of forward primer (10 μM), 1 µL of reverse primer, 5 μL of 10 × EasyTaq Buffer (TransGen Biotech, Beijing, China), 0.5 μL of EasyTaq DNA Polymerase (TransGen Biotech), 4 μL of 2.5 mM dNTPs, 1 μL of cDNA template and 37.5 μL of distilled water. The PCR conditions consisted of initial denaturation at 94 °C for 5 min, followed by 35 cycles of denaturation at 94 °C for 30 s, annealing at 68 °C for 30 s, extension at 72 °C for 1 min, and final extension at 72 °C for 5 min. The PCR products corresponding to the expected molecular size were extracted from the gels and purified using a StarPrep Gel Extraction Kit (Kangrun Biotech, Guangzhou, China), according to the manufacturer’s instructions. Both the PCR products and the expression vector pHT43 (MoBiTec GmbH, Oberhausen, Germany) were digested with restriction enzymes *Xba* I and *Sma* I (ThermoFisher Scientific, Waltham, MA, USA), then linked with T4 DNA ligase (Thermo Fisher) at 22 °C overnight. Consequently, the recombinant plasmid pHT43-ech42 was constructed and transformed into *E. coli* DH5α. The plasmid DNA was isolated and verified by DNA sequencing (Huada, Shenzhen, China).

### 4.3. Transformation of ech42 Gene to B. amyloliquefaciens

*B. amyloliquefaciens* was cultured at 37 °C in growth medium (1% peptone, 0.5% yeast extract, 1% NaCl and 9.1% sorbitol) to a final optical density of 0.85. The cell culture was cooled on ice for 10 min then carefully harvested by centrifugation at 5000× *g* and 4 °C for 5 min. The cells were washed four times with ice-cold electroporation medium (9% sorbitol, 9.25% mannitol and 10% glycerin), and suspended in the same solution. For transformation, the electro-competent cells were mixed with column-purified recombinant plasmid DNA and loaded into pre-chilled 1 mm gap electroporation cuvettes. After incubation for 2 min, the cell-DNA mixtures was shocked by 12.5 kv/cm, 15 kv/cm, 17.5 kv/cm, 20 kv/cm or 21 kv/cm voltage, respectively, using a GenePulserelectroporator (Bio-Rad GenePulserX cell) with the resistance set at 200 Ω, resulting in a time constant of 4.5–5.0 ms. Cells were then immediately diluted into 1 mL recovery medium (1% peptone, 0.5% yeast extract, 1% NaCl, 9% sorbitol and 7% mannitol) and incubated at 37 °C for 3 h to allow expression of the antibiotic resistant gene. Aliquots of the dilutions were then spread onto LB agar plates supplemented with 5 μg/mL chloramphenicol.

### 4.4. Detection the Growth Curves of B. amyloliquefaciens and the Recombinant Strain

The wild type *B. amyloliquefaciens,* and the recombinant strain (*B. amyloliquefaciens*-ech42), were inoculated in LB liquid medium with 5 μg/mL chloramphenicol. The growth rates of the two strains were tested by growing them in 100 mL LB liquid medium at 37 °C on a shaker (180 rpm). The optical density (OD) was measured every 2 h for 32 h using an RS232 PRINT spectrophotometer (Nanjing Kaidi High-Speed Analytical Company; Nanjing, China) at a wavelength of 630 nm. 

### 4.5. SDS-PAGE Assay

The molecular weight of the ech42 protein was detected by SDS-PAGE. The wild type *B. amyloliquefaciens,* and the recombinant strain *B. amyloliquefaciens*-ech42, were incubated in LB liquid medium at 37 °C with 1 mM of IPTG for 8 h. 0.4 mg/mL lysozyme (Coolaber, Beijing, China) was added in lysis of cells. Samples were boiled for 10 min with 2.5% (*w*/*v*) SDS. Proteins were separated by electrophoresis and gels were stained with Coomassie blue. The protein molecular weight marker (14,400) was used as a standard to calculate the molecular mass of target preteins.

### 4.6. Determination of Chitinase Activity

Chitinase activity was determined using the 3,5-dinitrosalicylic acid (DNS) assay. The quantity of reducing sugar was calculated based on comparison with a standard curve generated from known concentrations of *N*-acetylglucosamine (0–1 mg/mL). 1 mM of IPTG was added to the culture medium of *B. amyloliquefaciens*-ech42 and incubated on a rotary shaker (180 r/min) at 37 °C for 2, 4, 8, 12, and 24 h, respectively. Cells were centrifuged at 8000× *g*, 25 °C for 1 min and 500 µL of supernatant was added to 1.0 mL of 1% colloidal chitin (Solarbio, Beijing, China). The mixture was incubated at 37 °C for 1 h, and then terminated by the addition of 1.5 mL DNS (Coolaber, Beijing, China). The reaction was boiled in a water bath for 10 min then cooled to 25 °C. Next, the sample was centrifuged at 8000× *g* for 5 min and the volume was adjusted to 10 mL with distilled water. The absorbance was measured using a RS232 PRINT spectrophotometer (Nanjing Kaidi High-Speed Analytical Company; Nanjing, China) at 540 nm. The experiment was performed in triplicate.

### 4.7. Tomato Plant Growth Experiment

To test whether the *B. amyloliquefaciens*-ech42 strain has an enhanced effect against plant pathogens, *Botrytiscinerea*, which causes gray mold disease, was used in a greenhouse setting with tomato plants. Tomato plants, homozygous tomato variety 08016 (provided by the Tomato Research Institute of Northeast Agricultural University in China), were grown in a greenhouse at 25 °C/22 °C (day/night) with 16 h light and 8 h dark cycles for 12 weeks. The *Botrytiscinerea* strain was grown at 25 °C on a PDA plate. *Botrytiscinerea* spores were obtained from the surface of the 7-day old cultures and suspended in 5 mL of sterile distilled water containing 6.7 mMKH_2_PO_4_, 0.1 M glucose, 0.1% Tween, and pH 5, and then filtered through four layers of sterile cheesecloth to remove any adhering mycelia. Conidial suspensions were diluted to a concentration of 1 × 10^7^ spores mL^−1^ with sterile water as described above. Two experiments, prevention and disease treatment, were conducted in this study. In the prevention experiment, the 8 weeks old tomato seedlings were first treated with the wild type *B. amyloliquefaciens* or *B. amyloliquefaciens*-ech42 of concentration of 10^7^ CFU/mL by spraying with a water bottle. The plants were then treated with *Botrytiscinerea*s train after waiting for 24 h. For the treatment experiment, 8 weeks old tomato seedlings were first treated with *Botrytiscinerea* for 24 h. The seedlings were then sprayed with the wild type or the recombinant *B. amyloliquefaciens*. The control seedlings were sprayed with sterile water. Ninety-three fruits were divided into three groups (wild type, recombinant and control), and the SOD and PPO activities of each group were measured. The disease severity of each group was recorded [23]. after 7 days of treatment using Gong and colleagues’ 9-grade scoring system based on the area of lesions on tomato leaves as follows: 0, no symptoms; 1, <6%; 3, 6–10%; 5, 11–25%; 7, 26–50%; 9, >50%. The biocontrol efficiency of each treatment was evaluated based on the disease index which was calculated using the following formula:

Disease index = [Σ(number of infected leaves × disease grade)/(total number of leaves × the highest disease grade)] × 100. Four replications were performed for each sample.

### 4.8. Defense-Related Enzyme (PPO and SOD) Activity Assays

PPO activity was measured according to the method used by Alamelumangai and Li [22,24], with minor modification.

To assay PPO activity, 1 g of fresh leaves was pulverized in 10 mL of buffer, containing 0.24 g polyvinyl polypyrrolidone (PVPP) and 0.1 M sodium phosphate (pH 7.0), in an ice bath. A total of 6 groups of leaves were collected, including leaves treated with the wild type and recombinant bacteria and control leaves (treated with water) from both the prevention and treatment experiments. The pulverized leaves were centrifuged at 10,000× *g* for 10 min at 4 °C, and the supernatant was used for enzyme activity assays. A 20 μL aliquot of the supernatant was mixed with 1 mL of a solution containing 0. 4 mL solution A (7.10 g Na_2_HPO_4_, 5.25 g Citric acid, 200 mL distilled water) and 0.1 mL solution B (2.76 g Catechol, 50 mL distilled water) and incubated for 10 min at 30 °C. PPO activity was measured using a spectrophotometer at 420 nm. One unit of PPO activity was defined as an increase in absorbance of 0.01.

To assay the superoxide dismutase (SOD) activity, 0.5 g of fresh leaves [25] were placed in a pre-cooled mortar, and 1 mL of phosphate buffer was gradually added to the grinding process. Additional buffer was added to make the total volume to 5 mL. The ground leaves were centrifuged at 10,000× *g* for 10 min at 4 °C. A 0.05 mL aliquot of the supernatant was mixed with 3.25 mL of the reaction solution (13 mM dl-Methionine, 75 µM Nitrobluetetrazolium, 10 µM EDTA-Na_2_ and 0.2 µM Riboflavin). Phosphate buffer was used as control. SOD activity was measured using a spectrophotometer at 560 nm. One unit of PPO activity is defined as an increase in absorbance of 0.01.

### 4.9. Statistical Analysis

Fisher’s least significant difference (LSD) test was used to compare the significance. A *p* value < 0.05 was considered statistically significant.

## Supplementary Materials

Supplementary materials can be found at http://www.mdpi.com/1422-0067/19/8/2221/s1.
