# Supplementary material for: The Endochitinase of Clonostachysrosea Expression in Bacillus amyloliquefaciens Enhances the Botrytis cinerea Resistance of Tomato
Source: Int J Mol Sci. 2018 Jul 30;19(8):2221. doi: 10.3390/ijms19082221 (PMC6121428; doi:10.3390/ijms19082221)
Supplement: Supplementary file 1 [file ijms-19-02221-s001.pdf]

Table S1. PCR Primer sequences**.**

Gene	Sequences (5' to 3')
ech42 forward	TATCTAGACGTGCCACTCCTCGTATGGAGG
ech42 reverse	CCCCCCGGGGGAGAGGCTGTTCTTGAT

Table S2. Bacterial and fungal strains used in this study**.**

Strain	Source
Bacillus amyloliquefaciens	Tomato Research Institute, Northeast Agricultural University
Clonostachys rosea	Tomato Research Institute, Northeast Agricultural University
E. coli DH5α	Tomato Research Institute, Northeast Agricultural University
Botrytis cinerea	Tomato Research Institute, Northeast Agricultural University
Colletotrichum coccodes	Tomato Research Institute, Northeast Agricultural University
Pythium aphanidermatum	Tomato Research Institute, Northeast Agricultural University
Sclerotinia sclerotiorum	Tomato Research Institute, Northeast Agricultural University
Fusarium oxysporum	Tomato Research Institute, Northeast Agricultural University
Fusarium verticillioide	Tomato Research Institute, Northeast Agricultural University

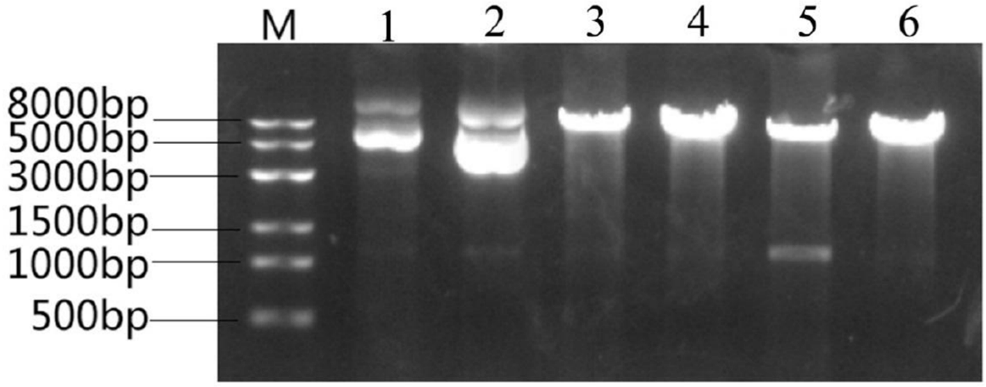

Figure S1. Construction and verification of vector pHT43-ech42 by single/double digestion. Lanes: M: Markers of molecular weight; 1. plasmid pHT43-ech42 without digestion; 2. plasmid pHT43. without digestion; 3. plasmid pHT43-ech42 digested with XbaI; 4. plasmid pHT43

digested with XbaI; 5.plasmid pHT43-ech42 digested with XbaI and SmaI; 6. plasmid pHT43 digested with XbaI and SmaI.

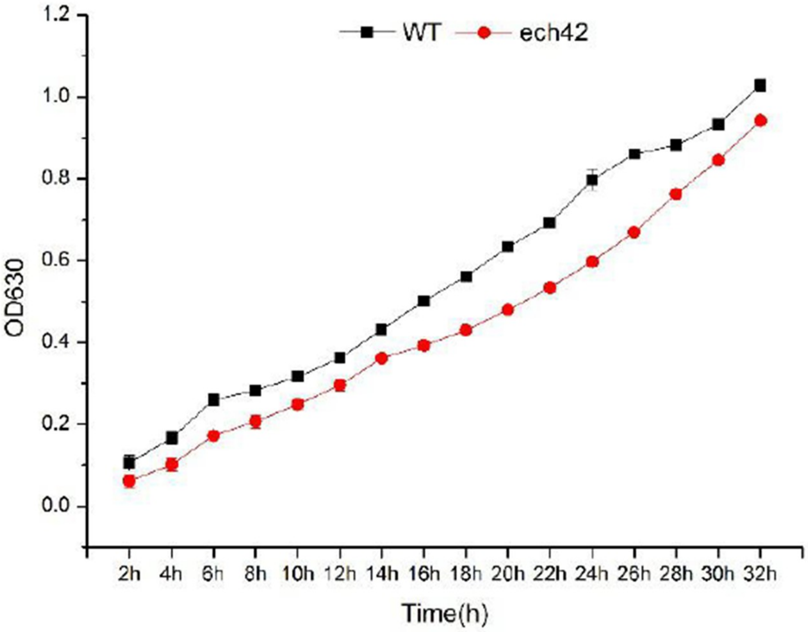

Figure S2. Growth curves of wild type *B. amyloliquefaciens* and *B. amyloliquefaciens*-ech42.
